# Extrapolation of imidacloprid toxicity between soils by exposing *Folsomia candida* in soil pore water

**DOI:** 10.1007/s10646-018-1965-x

**Published:** 2018-07-30

**Authors:** Afolarin O. Ogungbemi, Cornelis A. M. van Gestel

**Affiliations:** 1 Department of Ecological Science, Faculty of Science, Vrije Universiteit, Amsterdam, The Netherlands; 2 Institute for Environmental Sciences, Universität Koblenz-Landau, Landau, Germany; 3 Present Address: Department of Bioanalytical Ecotoxicology, UFZ-Helmholtz Centre for Environmental Research, Leipzig, Germany

**Keywords:** Collembola, Imidacloprid, Organic matter, Bioavailability, Soil ecotoxicology, Hazard/risk assessment

## Abstract

Soil properties like organic matter (OM) content show great variation, making it hard to predict the fate and effects of a chemical in different soils. We therefore addressed the question: can we remove the complexity of the soil matrix and yet accurately predict soil toxicity from porewater exposures? *Folsomia candida* was exposed to imidacloprid in natural (LUFA 2.2 [4.02% OM], Grassland [12.6% OM]) and artificial soils (OECD 5 [6.61% OM], OECD 10 [10.8% OM]), in pore water extracted from spiked LUFA 2.2 soil and in water. Toxicity decreased with increasing OM content except for Grassland soil, which had the highest OM content but the lowest clay content, suggesting a role of clay minerals in the binding of imidacloprid. Distribution coefficients for imidacloprid based on toxicity (Toxicity-*K*_d_) were derived by comparing effect concentrations in LUFA 2.2 soil and in water. Using these Toxicity-*K*_d_s to recalculate soil LC_50_s/EC_50_s to porewater concentrations, the differences in LC_50_/EC_50_s almost disappeared. The recalculated porewater LC_50_s did not differ by more than a factor of 0.55–1.43 from the LC_50_ obtained upon water exposure. This similarity suggests that the toxicity in the soil is dependent on porewater concentrations and can be obtained from water exposure. The porewater test and the corresponding “pore-water extrapolation concept” developed in this study may be used to predict the toxicity of chemicals in the soil and extrapolate among different soils.

## Introduction

In soil ecotoxicology, it is standard practice to expose soil organisms to chemicals in soil media, and the effect endpoint is usually estimated based on the concentration of the chemical in the soil. In the past, some limitations have been identified with this practice. First, exposure of organisms to the same chemical in different soils results in different effect concentrations (Van Gestel and Van Dis 1988; Dean-Ross 1983). Therefore, it is not possible to compare results of toxicity tests and this led to excessive testing of the same chemicals in different soils. Physicochemical properties such as organic matter (OM) content, clay content and pH are known to influence the fate and bioavailability of a chemical in the soil (Bonmatin et al. 2015). Hence, the fraction of chemical available for uptake by organisms varies, leading to different effect concentrations in different soils (Van Gestel 2012). This limitation was circumvented by developing standard soil media such as the OECD artificial soil (OECD 1984) and LUFA soils (LUFA Speyer Germany), which may ensure comparability and reproducibility of toxicity data. The OECD soil is utilized for standard tests such as the Earthworm acute and reproduction toxicity tests (OECD 2004) and the Collembolan reproduction toxicity test (OECD 2009). However, due to a better representation of the natural ecosystem, the LUFA soils are gaining wider acceptance.

Second, soft bodied and in-soil dwelling organisms such as earthworms and springtails are known to take up chemicals via the soil pore water (Belfroid et al. 1996; Fountain and Hopkin 2005). Hence, chemicals dissolved in the pore water are assumed to be highly bioavailable compared to those adsorbed to the soil. Although estimating the effect of chemicals on these organisms based on total soil concentrations is widely accepted, such estimates are quite unrepresentative of the actual concentrations causing the effect (Bradham et al. 2006). Appropriate knowledge of exposure levels is required for a proper estimate of ecotoxicological effects (Van Gestel and Weeks 2004). Of course, the internal concentration of a chemical in an organism would provide a better estimate of effects; however, measuring such concentrations is time and resource demanding and therefore unsuitable for routine ecotoxicological tests (Escher and Hermens 2004). Thus, it seems logical to advocate the use of porewater concentrations in soil ecotoxicological tests. For example, Van Gestel and Ma (1988) exposed earthworms to chlorophenols in four soils and recalculated the soil LC_50_ to porewater concentrations. They found a factor of 4.4–12.8 difference for soil LC_50_s, which significantly reduced to a factor of 1.2–2.4 when LC_50_s were based on porewater concentrations. Consequently, they postulated the porewater hypothesis which states that the concentration of a chemical in an organism is considered to be related to the porewater concentration which in turn depends on the sorption behavior of the chemical in the soil.

The porewater hypothesis has been confirmed by other studies that reported greatly reduced variation between soils when employing porewater concentrations (Ronday et al. 1997; Martikainen and Krogh 1999; Waalewijn-Kool et al. 2014). The validity of the porewater hypothesis motivates the extrapolation of toxicity data from one soil to another as well as predicting soil toxicity from water exposure. It therefore is surprising that the simplification offered by this hypothesis has not been utilized to a greater extent in soil ecotoxicology. However, some exceptions exist for the implementation of this hypothesis (Van Gestel 1997). First, highly lipophilic chemicals are expected to strongly adsorb to the soil matrix leading to increased importance of oral uptake. Belfroid (1994) found that the contribution of oral uptake increased when earthworms were exposed to highly lipophilic chemicals in soils with high organic matter content. Similarly, Jager et al. (2003) showed the importance of oral uptake after exposing earthworms to hydrophobic chemicals while preventing them from feeding. Both authors concluded that the contribution of the oral uptake route is less than a factor of 2 and the porewater hypothesis could still be useful in risk assessment. Second, for metals, sorption is governed by other factors including pH and clay content. Therefore an alternative hypothesis was proposed, which relates toxicity to free metal ion activity in the soil solution (Van Gestel 1997). This hypothesis has been elaborated in the Biotic Ligand Model (see e.g., Ardestani et al. 2014).

Ronday and Houx (1996) assessed the suitability of exposing seven species of soil organisms in simulated pore water and *Folsomia candida* was considered to be most suitable and sensitive. Hence, *F. candida* was chosen as the organism for the present study because of its vulnerability to soil contamination via soil pore water, which makes it a good soil quality indicator (Filser et al. 2014). Springtails are equipped with a ventral tube which enables them to exchange water and oxygen with the environment (Lock and Janssen 2003; Fountain and Hopkin 2005). Their hydrophobic ventral tube and ability to walk on water make *F. candida* suitable for aquatic toxicity tests (Houx et al. 1996). In the present study, we aimed at answering the following questions: (1) To what extent do differences in soil organic matter content influence the toxicity of imidacloprid to *F. candida*? (2) Can we remove the complexity of the soil matrix and yet accurately predict soil toxicity from porewater exposures? Based on these questions, we hypothesized that the toxicity obtained on exposure to organic chemicals in water would equal the toxicity obtained in pore water extracted from spiked soil, and this should correlate with the toxicity obtained in the soil via factoring in organic matter content. In view of this, we exposed *F. candida* in soil, water and pore water extracted from spiked soil.

## Materials and methods

### Test organism

*Folsomia candida* (Denmark strain, Vrije Universiteit, Amsterdam) were cultured in plastic boxes with a moist bottom made of a mixture of plaster of Paris and charcoal at a ratio 10:1. Animals were fed dried baker’s yeast (Instant yeast from Algist Bruggeman N.V, Ghent, Belgium) and cultures were maintained at 20 °C and 12 h light/12 h dark cycle in a climate chamber. Adult *F. candida* were incubated in culture boxes and removed after 2 days of egg laying. Juveniles emerged after 10 days and the toxicity tests were initiated with 10–12 day old animals.

### Soil spiking with imidacloprid

This study used two OECD artificial soils, LUFA 2.2 standard soil (LUFA-Speyer 2.2 Sp 2121, Germany) and a Grassland soil collected in the Netherlands (Natal-da-Luz et al. 2012). The first artificial soil was prepared by mixing 70% silver sand, 20% kaolin clay and 10% peat, hereafter referred to as OECD 10. The second artificial soil was made up of 75% silver sand, 20% kaolin clay and 5% peat, hereafter referred to as OECD 5. Both artificial soils were prepared according to OECD (2009). LUFA 2.2 soil (7.7% clay, 4.02% OM) was purchased and dried in the oven at 50 ^o^C prior to use. Grassland soil (4.8% clay, 12.6% OM) was collected in 2012 from a field site located in the Netherlands and has since been stored in dry form in the laboratory.

The Imidacloprid powder (98% purity) used for the toxicity tests was obtained from Bayer Crop Science, Germany in 2014. Imidacloprid has a low vapor pressure and Henry’s constant of 1.0 × 10^−7^ mm Hg and 6.5 × 10^−11^ atm m³/mole, respectively, indicating low volatility. It has a relatively high water solubility (0.51 g/L) and low octanol-water partition coefficient [Log Kow = 0.57] (Fossen 2006). Solutions of imidacloprid were prepared in deionized water to give concentrations of 45.5, 36.6, 45.5 and 55.6 mg/L for the stocks to spike LUFA2.2, Grassland, OECD 10 and OECD 5 soil, respectively. The solutions were serially diluted by a factor of 3 and spiked into the test soils to obtain nominal soil concentrations of 0.03, 0.11, 0.33, 1.11, 3.33 and 10 mg/kg dry soil. The concentrations in the stock solutions and the dilutions were undertaken in such a way that the obtained volume was also sufficient to moisten the soil to the desired level. Control soil was spiked with deionized water only. LUFA 2.2, OECD 5 and OECD 10 soils were moistened to 50% of the water holding capacity (WHC) while Grassland soil was moistened to 38% of the WHC. The spiked soils were allowed to stand overnight for appropriate equilibration of the chemical.

### Soil toxicity test

The effect of imidacloprid on the survival and reproduction of *F. candida* was investigated according to OECD (2009). Ten (10–12 day old) juvenile *F. candida* were exposed in 100 mL glass jars containing 30 g of the test soil spiked with varying concentrations of imidacloprid (0.03, 0.11, 0.33, 1.11, 3.33 and 10 mg/kg dry soil) and a control. Five replicates per concentration were prepared and a few grains of dried baker’s yeast were added for food. The glass jars were aerated and remoistened weekly to replenish air and water, respectively. According to Crouau and Cazes (2003), variability of the reproduction test can be reduced by increasing the duration of the test. Therefore, survival and reproduction were assessed after 14 (for LUFA 2.2 only) and 33 days (for the four test soils) of incubation by extracting the animals from the soil by means of flotation (OECD 2009). Photographs were taken to manually count the number of juveniles and adults with image J software.

### Porewater toxicity tests

After overnight equilibration of LUFA 2.2 soil spiked as described above, soil samples were collected and centrifuged with a relative centrifugal force of 2000×*g* over a Schleicher and Schuell 0.45 μm membrane filter placed in between two filter papers in a centrifuge (MSE FALCON 6/300). The resulting filtrate was collected and utilized as the porewater exposure media.

To enable comparison of exposures in the porewater media with effects following exposure to water media with known concentrations, water media were prepared by dissolving an appropriate amount of imidacloprid in deionized water to give a concentration of 30 mg/L. This solution was further diluted with deionized water to give nominal concentrations of 0.03, 0.11, 0.33, 1.11, 3.33, 10 and 30 mg/L, and including a water control.

*F. candida* (10–12 day old) were simultaneously exposed to imidacloprid in pore water extracted from spiked LUFA 2.2 soil (PW_e_) and in water for 33 days, following a modification of the test method reported by Houx et al. (1996). One mL of the respective solution was transferred into small glass vials (volume of 2 ml and dimensions of 12 × 35 mm) arranged in a multi-well column. Four *F. candida* were added to the solution in each vial and the glass vials were loosely closed. Five replicates per treatment were prepared and food was not added. Assessments were done daily by recording the number of active, affected, moribund or dead animals. Animals were judged dead if immobile after disturbing with a wave of air. Additionally, behavioral effects were monitored daily by categorizing the activity of the organisms. The effects were categorized according to Houx et al. (1996) as: (1) hyperactivity and escape from the water surface to the glass wall; (2) vigorous shaking of the body; (3) spring-forks folded out so that jumping was impossible; (4) standing upright or lying sideways with vigorous flex-and-stretch movements of the body and energetic movements of legs and antennae; (5) standing or lying with trembling legs and antennae; (6) droplets of clear colorless fluid emerging from all parts of the body; (7) standing or lying with hardly noticeable shivering in legs and antennae; (8) dead animals were fully stretched out, with their fork folded out and their legs swollen. These behavioral effects were aggregated into four groups during the assessments: Active (effect 1), Affected (effects 2, 3 and 4), Moribund (effects 5, 6 and 7) and Dead (effect 8).

### Data analysis

Results were obtained as numbers of surviving adults/juveniles and a dose-response curve was fitted to the data using a log-logistic or a Weibull model (Ritz and Streibig 2005). The lethal concentration at which 50% of the adults were killed (LC_50_) was estimated from the dose-response curve. The best fitting curve was selected based on Akaike information criteria. Additionally, results were obtained as number of active and affected adults for the soil, porewater and water tests. In these cases, lethality and moribundity were combined as the end point (LMC_50_). The concentration of imidacloprid in the PW_e_ could not be determined because of the large volume of the pore water that was required for the analysis. Therefore, porewater effect concentrations had to be derived indirectly from water effect concentrations by correlating PW_e_ LMC_50_ (mg/kg soil) against water LMC_50_ (mg/L). All statistical analyses related to effect concentrations were implemented using the statistical software environment R for Windows (RStudio Team 2016).

To estimate porewater effect concentrations for the other soils, soil LC_50_/EC_50_s were recalculated to give modelled porewater (PW_m_) LC_50_/EC_50_s. This recalculation was facilitated by determining the binding of imidacloprid to soil. For that purpose, the distribution coefficient (*K*_d_) based on toxicity (Toxicity-*K*_d_) was estimated for imidacloprid by dividing the soil LMC_50_ with the water LMC_50_ (Eq. 1).

1 $$LUFA\,2.2\,Toxicity-K_{d} = \frac{{soil\,LMC_{50}}}{{water\,LMC_{50}}}$$

An organic carbon partition coefficient (*K*_oc_) for imidacloprid in LUFA 2.2 soil was estimated by dividing the Toxicity-*K*_d_ with the fraction of organic carbon (F_oc_) in the soil (Eq. 2).

2 $$K_{oc} = \frac{{LUFA\,2.2\,Toxicity - K_{d}}}{{LUFA\,2.2\,F_{oc}}}$$

Imidacloprid is assumed to mainly bind to OM and its *K*_oc_ was found to remain similar in different soils (Liu et al. 2006). We then used the LUFA 2.2 *K*_oc_ based on toxicity as a normalizing factor to estimate Toxicity-*K*_d_ values for the other soils (Eq. 3).

3 $$Toxicity - K_{d} = K_{oc} \times F_{oc}$$

The estimated Toxicity-*K*_d_s were used to recalculate soil LC_50_/EC_50_s in mg/kg dry soil into PW_m_ LC_50_/EC_50_s in mg/L for the four different test soils (Eq. 4).

4 $$LC_{50}\,mg{\mathrm{/}}L = \frac{{LC_{50}\,mg/kg}}{{Toxicity - K_{d}}}$$

## Results

### Control performance

The toxicity tests in the four different soils were considered valid according to the validity criteria stipulated by OECD (2009). Mean adult mortality in the controls did not exceed 20% with values of 16, 4, 12 and 8% for LUFA 2.2, OECD 5, OECD 10 and Grassland soils, respectively. The mean number of juveniles in the control vessels met the required minimum of 100 with average values of 612, 694, 564 and 394, respectively. The coefficients of variation for the juvenile numbers in replicate controls were below 30% with values of 17.8, 12.4, 26.1 and 19.7% for LUFA 2.2, OECD 5, OECD 10 and Grassland soils, respectively. Cumulative control mortalities in the porewater and water tests were 5 and 0%, respectively, hence the tests were considered valid. Even though animals were not fed in porewater and water tests, they showed good fitness in the controls for the duration of the test. However, activity of the animals was lower in porewater controls than in water controls.

### Toxicity estimations in soil, water and pore water

**Fig. 1:**
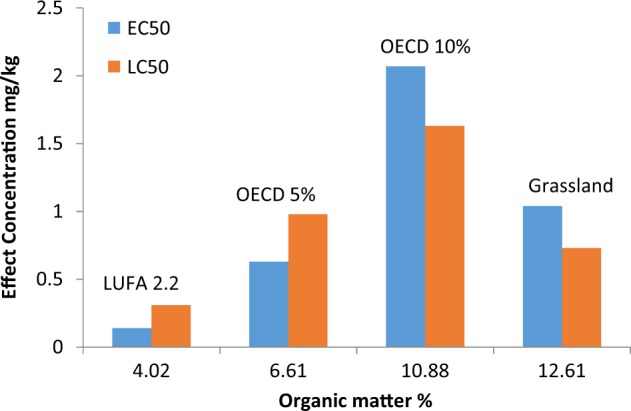
Bar chart showing the relationship between effect concentrations (LC_50_ and EC_50_) for the toxicity of imidacloprid to *Folsomia candida* and the organic matter content of the four test soils

Imidacloprid affected both adult survival and reproduction of *F. candida* in a dose-related manner (Figure S1–S4, Supporting Information). The estimated LC_50_ and EC_50_ values are reported in Table 1. Toxicity of imidacloprid to *F. candida* increased with decreasing OM content except for Grassland soil which had the highest OM content. Imidacloprid was most toxic in LUFA 2.2 which is the soil with the lowest OM content (LC_50_ = 0.31, EC_50_ = 0.14 mg/kg dry soil) and least toxic in OECD 10 (LC_50_ = 1.63, EC_50_ = 2.07 mg/kg dry soil). The relationship between effect concentrations and OM content (Fig. 1) without Grassland soil resulted in Pearson correlation coefficients (r) of 0.98 and 0.99 for LC_50_ and EC_50_, respectively.

**Table 1:** Properties of four different soils and the corresponding LC_50_/EC_50_ values for the toxicity of imidacloprid to *Folsomia candida* exposed for 33 days in these soils

Soil	pH CaCl_2_	OM content (%)	WHC^c^ (%)	Clay content (%)	LC_50_ (mg/kg dry soil)	EC_50_ (mg/kg dry soil)
LUFA 2.2	6.26	4.02 ± 0.05	44^a^	7.7^a^	0.31 (0.22–0.39)	0.14 (0.11–0.17)
OECD 5	6.05	6.61 ± 0.09	32^c^	20	0.98 (0.63–1.34)	0.63 (0.34–0.92)
OECD 10	5.92	10.9 ± 0.17	40^c^	20	1.63 (0.96–2.30)	2.07 (1.42–2.72)
Grassland	6.84	12.6 ± 0.79	73^b^	4.8^b^	0.73 (0.35–1.11)	1.04 (0.80–1.27)

LC_50_, effects on survival; EC_50_, effects on reproduction; *OM*, organic matter (±SD, *n* = 3) determined as loss on ignition at 500 °C; *WHC*, water-holding capacity; LC_50_/EC_50_ (95% confidence interval in parentheses)

^a^Data obtained from LUFA Speyer analyses data sheet

^b^Data from Natal-da-luz et al. (2012)

^c^WHC determined as moisture content after saturation with water

**Fig. 2:**
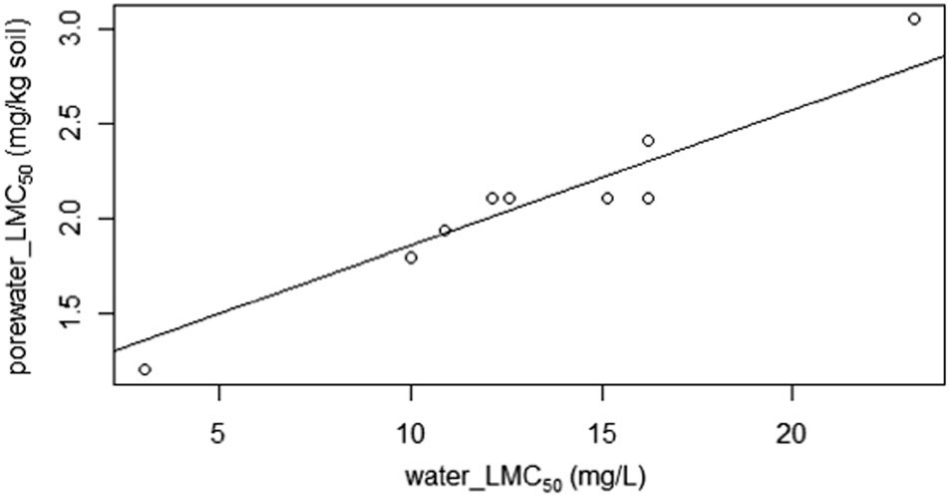
Regression curve (R^2^ = 0.89, y = 0.085× + 0.98) relating LMC_50_s for the toxicity of imidacloprid to *Folsomia candida* exposed to pore water extracted from freshly spiked LUFA 2.2 soil to LMC_50_s for exposure in water. LMC_50_s in pore water are in mg/kg dry soil, those in water in mg/L. Data points show LMC_50_s recorded at different times of exposure

**Fig. 3:**
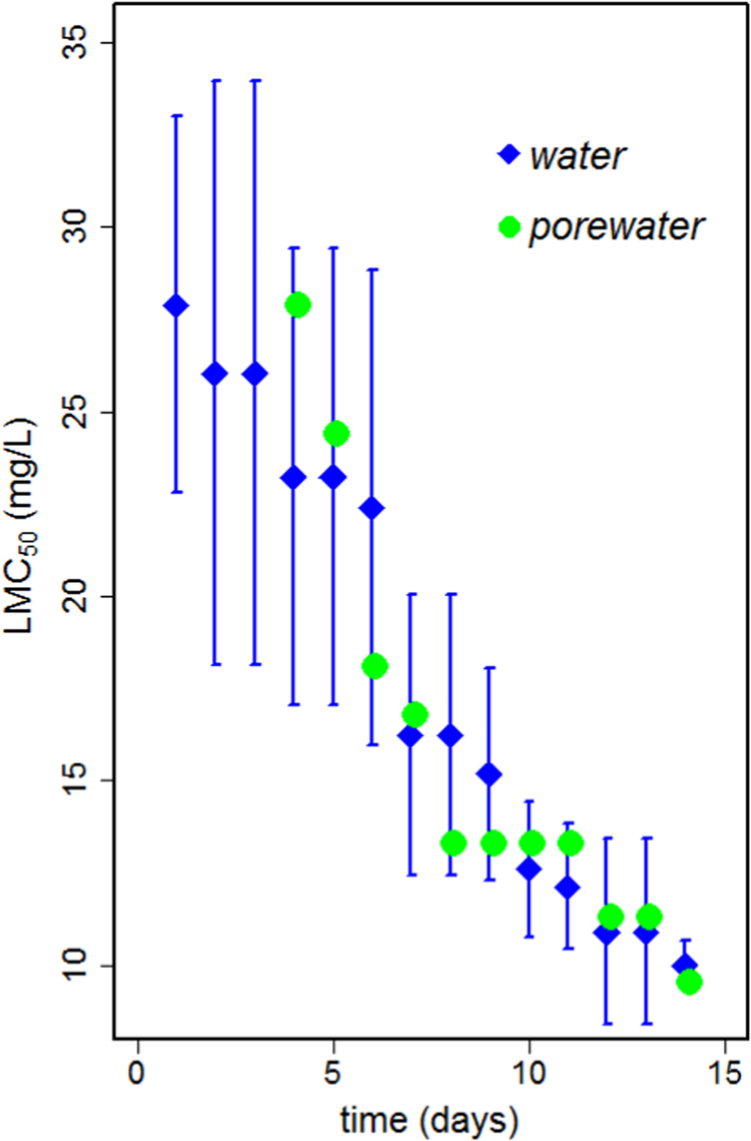
Plot of LMC_50_s against exposure time for the toxicity of imidacloprid to *Folsomia candida* exposed in water or in pore water extracted from freshly spiked LUFA 2.2 soil. Points show the estimated LMC_50_s in mg/L and error bars show the 95% confidence intervals for water LMC_50_s only. Predicted porewater LMC_50_s are within the confidence limits of water LMC_50_s. See Table 1, Supporting Information for all values with corresponding confidence intervals

For the porewater and water tests, the animals did not quickly experience death but did show behavioral effects. Generally, there was a progression of these behavioral effects (Active-Affected-Moribund-Dead) during the course of exposure until death. The Lethal-moribund effect concentrations (LMC_50_) for porewater and water tests are reported in Table S1 in the Supporting Information and the LMC_50_s estimated for soil toxicity are in Table 2. Since concentration of imidacloprid in the pore water extracted from spiked LUFA 2.2 soil (PW_e_) was not measured, PW_e_ LMC_50_s in mg/kg dry soil were converted to values in mg/L by comparison with the water LMC_50_s in mg/L. This comparison resulted in a regression with an R^2^ of 0.89, Y = 0.085× + 0.98 (Fig. 2). This conversion was based on the assumption that the response of *F. candida* to imidacloprid in PW_e_ and water should be similar since sorption to dissolved organic matter may not be influential (Houx and Aben 1993). The estimated LMC_50_s for PW_e_ were very similar to the water LMC_50_s except for exposure days 2 and 3, where the LMC_50_s were about 3 fold higher for PW_e_ than for water exposure. Figure 3 shows decrease of the LMC_50_s over time.

**Table 2:** Toxicity-*K*_d_ values describing the sorption of imidacloprid in LUFA 2.2 soil estimated from the LMC_50_s for lethal and moribund effects on *Folsomia candida* exposed for 14 and 33 days in soil, in water, and in pore water extracted from the LUFA 2.2 soil

Time (d)	Soil LMC_50_ (mg/kg soil)	Water LMC_50_ (mg/L)	Porewater LMC_50_ (mg/kg soil)	Predicted Porewater LMC_50_ (mg/L)	Toxicity-*K*_d_ (L/kg)
14	0.28 (8.26)	9.99 (24.4)	1.79 (5.55)	9.53 (53.8)	0.03
33	0.16 (0.31)	3.04 (6.22)	1.20 (1.38)	2.59 (4.71)	0.05

LC_50_s are in parentheses. Predicted porewater LMC_50_s and LC_50_s were estimated from a correlation of water and extracted porewater toxicity values; see Fig. 1

The distribution coefficient for the binding of imidacloprid to LUFA 2.2 soil (F_oc_ = 0.023) estimated from the *K*_ow_ of imidacloprid (3.7) is 0.085 L/kg. The toxicity-based distribution coefficients (Toxicity-*K*_d_) estimated from the LMC_50_s for the toxicity to *F. candida* in soil and water after 14 and 33 days of exposure (Table 2) were similar at 0.03 L/kg and 0.05 L/kg, respectively. Using the Toxicity-*K*_d_ for day 33, an organic carbon partition coefficient (Toxicity-*K*_oc_) of 2.3 L/kg was estimated for imidacloprid in LUFA 2.2 soil.

**Fig. 4:**
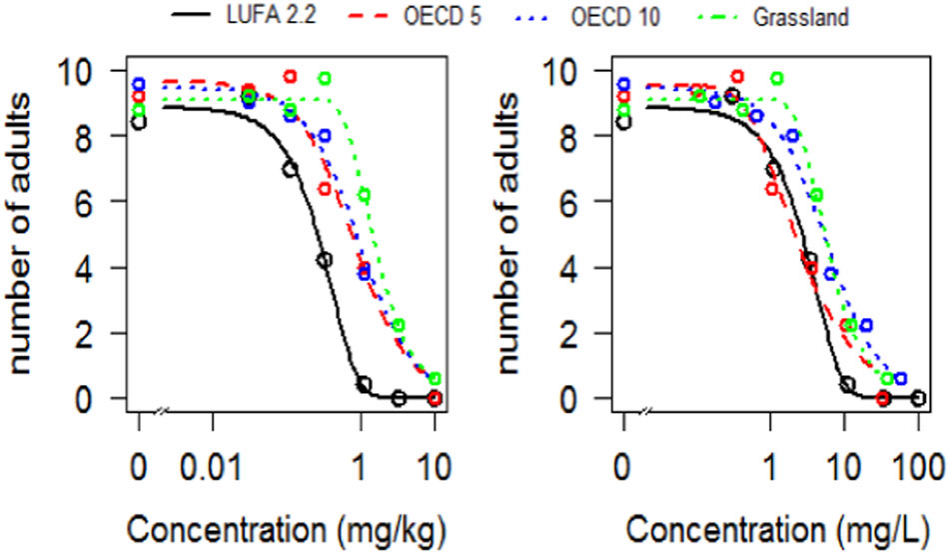
Dose response relationships for the toxicity of imidacloprid to *Folsomia candida* in four different soils. Left: dose-response curves for the effects on survival based on concentrations of imidacloprid in mg/kg dry soil. Right: dose-response curves based on recalculated imidacloprid concentrations in pore water. Decreased variability can be seen in the curves estimated in mg/L

Since the variability of imidacloprid *K*_d_s in different soils was greatly reduced after normalizing for OM content (Liu et al 2006), the Toxicity-*K*_oc_ for imidacloprid obtained was then used to estimate Toxicity-*K*_d_s for imidacloprid in the other three test soils (Table 3). When these Toxicity-*K*_d_s were used to recalculate the LC_50_s/EC_50_s in mg/kg dry soil into modelled porewater (PW_m_) LC_50_s/EC_50_s in mg/L, the differences between the four test soils became substantially lower (Table 3). Factor differences of 5.2 (mg/kg dry soil) and of 2.6 (mg/L) were calculated for LC_50_s, and of 15 (mg/kg dry soil) and 5.4 (mg/L) for EC_50_s. A graphical representation of the decrease in variation in LC_50_s obtained after conversion to mg/L can be seen in Fig. 4.

**Table 3:** Toxicity-K_d_ for the sorption of imidacloprid and modelled porewater LC_50_ values (in mg/L) for its toxicity to *Folsomia candida* in four different soils

Soil	OC content (%)	Toxicity-*K*_d_ (L/kg)	LC_50_ (mg/kg dry soil)	LC_50_ (mg/L)	EC_50_ (mg/kg dry soil)	EC_50_ (mg/L)
LUFA 2.2	2.32	0.053	0.31	5.85	0.14	2.64
OECD 5	3.82	0.088	0.98	11.1	0.63	7.16
OECD 10	6.29	0.145	1.63	11.2	2.07	14.3
Grassland	7.29	0.168	0.73	4.35	1.04	6.19

%*OC* = organic carbon content (estimated by dividing OM% with 1.73)

## Discussion

### Reliability of the toxicity-Kd

In the present study, a toxicity-*K*_d_ based on the LMC_50_s for the toxicity of imidacloprid to *F. candida* in soil and water was estimated (Table 2). The toxicity-*K*_d_ was considered to be reliable because the value estimated via different methods proved to be similar; (1) Toxicity-*K*_d_s estimated for exposure durations of 14 (0.03 L/kg) and 33 days (0.05 L/kg) are similar, (2) the 33-day-Toxicity-*K*_d_ is similar to the Literature-*K*_d_ of 0.085 L/kg estimated from the *K*_ow_ of imidacloprid, and (3) the 33-day-Toxicity-*K*_d_ is similar to the slope (0.085 L/kg) of the water-porewater regression curve which also represents a toxicity distribution. Furthermore, the LC_50_ (0.31 mg/kg dry soil) and EC_50_ (0.14 mg/kg dry soil) obtained for LUFA 2.2 soil in the present study is consistent with the values (LC_50_ = 0.47 mg/kg; EC_50_ = 0.26 mg/kg dry soil) obtained by De Lima e Silva et al. (2017) and the ones (LC_50_ = 0.44 mg/kg; EC_50_ = 0.29 mg/kg dry soil) obtained by Van Gestel et al. (2017). Similarly, the LC_50_ (1.63 mg/kg dry soil) found for OECD 10 soil is consistent with the value of 1.38 mg/kg dry soil reported by Reynolds (2008). This shows the reproducibility of the results from this study and hence, the validity of soil toxicity values used to estimate toxicity-*K*_d_. Even though the toxicity-*K*_d_ seems valid, the occurrence of discrepancies cannot be ruled out. For instance, the polarity of imidacloprid suggests that its sorption to soil may not only be governed by partitioning to organic matter but also by the interaction with clay minerals (Rutherford et al. 1992). Moreover, the toxicity-*K*_oc_ of 2.3 L/kg estimated for imidacloprid in LUFA 2.2 soil does not agree with literature *K*_oc_ values of 132–300 L/kg (Fossen 2006; Liu et al. 2006). This discrepancy could be due to the chemical-based method used to determine the literature *K*_oc_ values compared to the toxicity-based method employed in the present study. Additionally, LUFA 2.2 soil was not included in the range of soils used to derive the *K*_oc_ values reported in the literature.

### Predicting toxicity 1: comparing toxicity in soil, water and pore water

The extent of absorption, distribution, metabolism, excretion and hence the consequent toxicity of a chemical, is highly dependent on the exposure route of the organism (Fu et al. 2013). Several studies have shown that pore water is the most important route of chemical exposure for in-soil dwelling organisms by correlating toxicity to porewater concentrations (Martikainen and Krogh 1999; Styrishave et al. 2010). For that reason, uptake via pore water and water was investigated in this study and an attempt was made to predict soil toxicity. After estimating the Toxicity-*K*_d_ from LMC_50_s, we then proceeded to use this value to estimate a different toxicity endpoint (LC_50_s). Interestingly, the modelled porewater (PW_m_) LC_50_s recalculated from the soil LC_50_s of 4.35–11.2 mg/L only differed by a factor of 0.55–1.43 from the value of 6.22 (CI = 4.95–7.50) mg/L obtained upon exposure in water. Similarly, the recalculated PW_m_ LC_50_ for LUFA 2.2 soil (5.85 mg/L) corresponds to the value (4.71 mg/L) obtained upon exposure in pore water extracted from LUFA 2.2 soil (PW_e_) (Tables 2 and 3). Ronday et al. (1997) also found similar toxicity relationships for the exposure of *F. candida* to carbofuran in water and pore water (using recalculated porewater values).

These comparisons imply that the toxicity of a chemical in the soil is mainly dependent on its porewater concentration and can be obtained from water exposures. This led to the postulation of a new hypothesis referred to as the “porewater extrapolation concept”, which states that the toxicity of a chemical in the soil to *F. candida* can be related to porewater toxicity using the Toxicity-*K*_d_, and this porewater toxicity is considered to be equal to the water toxicity (Figure S5, Supporting Information). Applying this concept to toxicity data obtained in the present study means that the toxicity in the four soils can be predicted by exposing animals in water. Considering that the obtained water toxicity is equal to porewater toxicity, this can then be converted to soil toxicity values using the toxicity-*K*_d_. Most importantly, in cases where toxicity data is required for different soils, obtaining a single water toxicity data and *K*_d_s for different soils should be recommended. In such cases, water exposure is clearly a valuable and precise technique to predict soil toxicity. The “porewater extrapolation concept” could potentially be an extension of the porewater hypothesis and further research on the verification of such toxicity relationships is urgently required. However, there are indications that the porewater hypothesis, and hence the porewater extrapolation concept, may not be valid for highly lipophilic chemicals (Log K_ow_ > 5) which are likely to be taken up via the oral route (Belfroid et al. 1996; Jager et al. 2003).

### Predicting toxicity 2: comparing toxicity in different soils

The porewater hypothesis proposed by Van Gestel and Ma (1988, 1990) assumes that the concentration of the chemical in the pore water causing a certain level of biological effect should be similar for different soils, and independent of the OM content (or other properties) of the soils. The availability of *K*_d_ values therefore allows the extrapolation of toxicity data between different soils (van Gestel 1997). In the present study, after recalculating the soil LC_50_ values, similar porewater LC_50_s were obtained for both OECD artificial soils (OECD 5 = 11.1 mg/L, OECD 10 = 11.2 mg/L), and these values were about a factor of 2–2.5 higher than those for the two natural soils [LUFA 2.2 = 5.85 mg/L, Grassland = 4.35 mg/L] (Table 3). However, the disparity between artificial and natural soils was not observed when the EC_50_ values were recalculated and this is probably due to imidacloprid acting specifically on adult survival rather than reproduction (De Lima e Silva et al. 2017). Martikainen and Krogh (1999) also found only slight deviations for recalculated water LC_50_s for the toxicity of dimethoate to *F. candida* in different soils, and they attributed the deviations to differences in microbial activity. This conclusion cannot be made in the present study because neither microbial activity nor degradation rates were monitored. An earlier study, however, showed imidacloprid to be fairly stable in LUFA 2.2 soil, with estimated half-lives of > 125 days (Van Gestel et al. 2017). The differences between the LC_50_s for artificial and natural soils could be due to different types of organic matter (peat) and clay (kaolin clay) present in the OECD artificial soils compared to the natural soils. These artificial soils also contained 65% more clay than the natural soils and therefore sorption to clay could not be neglected. Large specific surface area and high cation exchange capacity (CEC) make clay particles excellent adsorbents especially for cationic chemicals (Bhattacharyya and Gupta 2008). This suggests that the higher clay content of the OECD soils could lead to higher sorption of polar organic compounds such as imidacloprid. This form of sorption is not accounted for by measuring OM content, and might explain for the differences in recalculated water LC_50_ values (Ronday et al. 1997). Despite having the highest OM content, the low clay content of Grassland soil could be a plausible explanation for its relatively higher soil toxicity. Surprisingly, Grassland soil also had the highest toxicity after recalculating to porewater LC_50_ values (Table 3). As reported by Natal-da-Luz et al. (2012), this higher toxicity may be attributed to the presence of (low concentrations of) Lead (34.9 mg/kg), Zinc (36.2 mg/kg) and polycyclic aromatic hydrocarbons (1.33 mg/kg) in the Grassland soil.

## Conclusion

The present study examined the role of porewater exposure in mediating the toxicity of imidacloprid to the springtail *F. candida*. We showed that clay minerals, if present in relatively high amount, can potentially influence the bioavailability of imidacloprid in the soil. Based on the porewater tests, we conclude that porewater and water exposures can potentially predict soil toxicity. Comparison of different exposure media enabled the postulation of the “porewater extrapolation concept,” which relates soil toxicity to porewater and water toxicity, respectively. This study also presents an easy estimation method for *K*_d_ values that are toxicity-relevant, and which proves to be useful in cases of financial, technical and resource limitations in chemical analysis. It is strongly advised to validate this method before application in practice.

Although the realism of exposing soil animals in water is not straightforward and visible, this study was able to show the relevance of this kind of exposure for not only predicting lethal effects in the soil but also estimating intrinsic toxicity of a chemical without the influence of soil sorption. We conclude that the porewater test developed in this study may be used to predict the toxicity of water soluble-organic chemicals in the soil, as well as to extrapolate toxicity data among different soils.

## Electronic supplementary material

Supplementary Information
