# Supplementary material for: Extrapolation of imidacloprid toxicity between soils by exposing Folsomia candida in soil pore water
Source: Ecotoxicology. 2018 Jul 30;27(8):1107–15. doi: 10.1007/s10646-018-1965-x (PMC6153508; doi:10.1007/s10646-018-1965-x)
Supplement: Supplementary file 1 — Supplementary Information [file 10646_2018_1965_MOESM1_ESM.docx]

**Supplementary Information to**

Extrapolation of Imidacloprid toxicity between soils by exposing *Folsomia candida* in soil pore water

Afolarin O. Ogungbemi^1,2,#^, Cornelis A. M. van Gestel^1*^

^1^Department of Ecological Science, Faculty of Science, Vrije Universiteit, Amsterdam, The Netherlands.

^2^Institute for Environmental Sciences, Universität Koblenz-Landau, Landau, Germany.

^#^Present address: AO, Department of Bioanalytical Ecotoxicology, UFZ-Helmholtz Centre for Environmental Research, Leipzig, Germany

*Corresponding author: [kees.van.gestel@vu.nl](mailto:kees.van.gestel@vu.nl), +31 20 59 87079


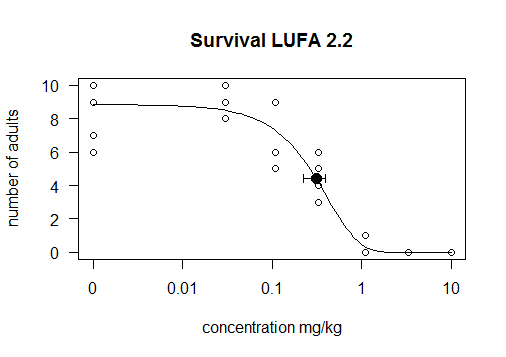


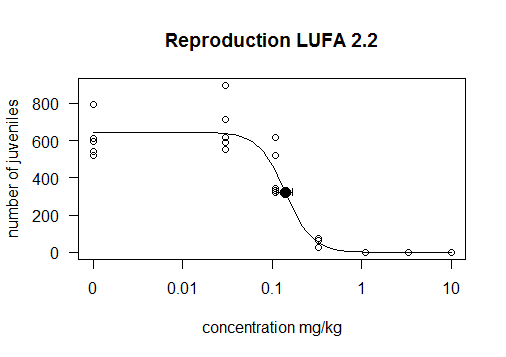


Figure S1: Dose-response relationships for the effect of imidacloprid on the survival (Top) and reproduction (Bottom) of *Folsomia candida* exposed in LUFA 2.2 soil for 33 days


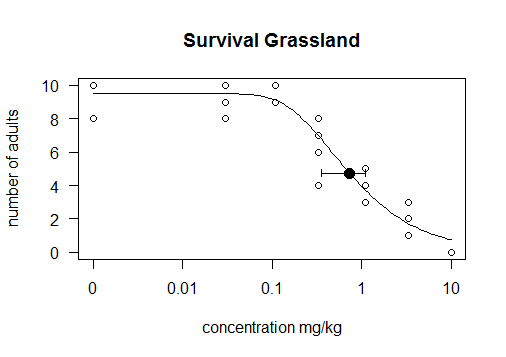


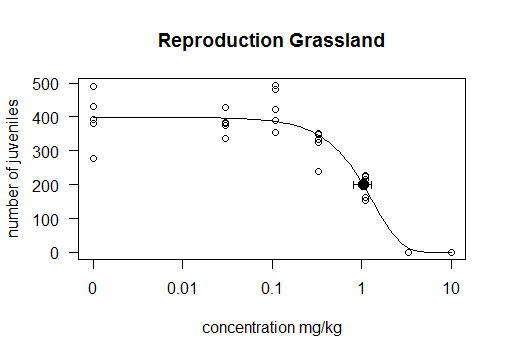


Figure S2: Dose-response relationships for the effect of imidacloprid on the survival (Top) and reproduction (Bottom) of *Folsomia candida* exposed in Grassland soil for 33 days


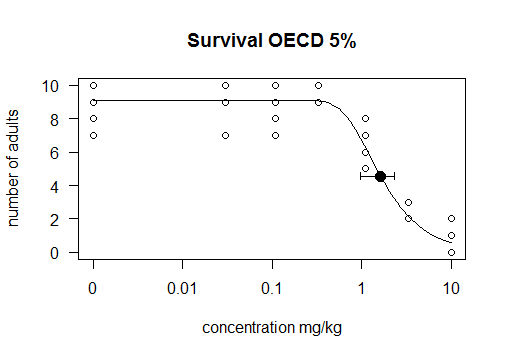


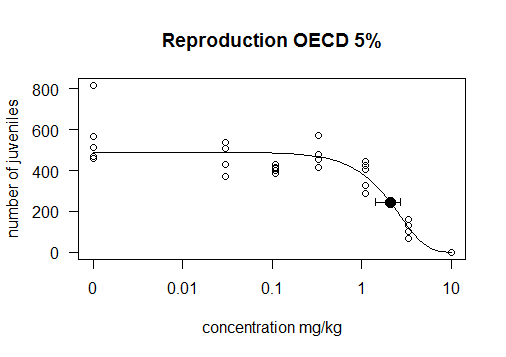


Figure S3: Dose-response relationships for the effect of imidacloprid on the survival (Top) and reproduction (Bottom) of *Folsomia candida* exposed in OECD 5 soil for 33 days


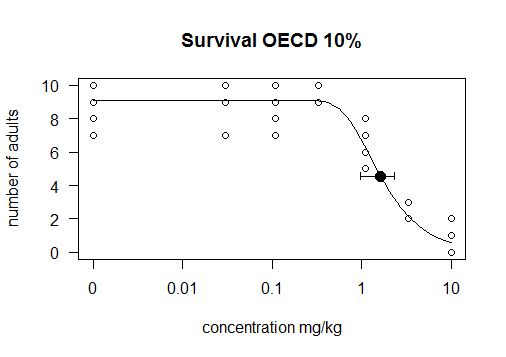


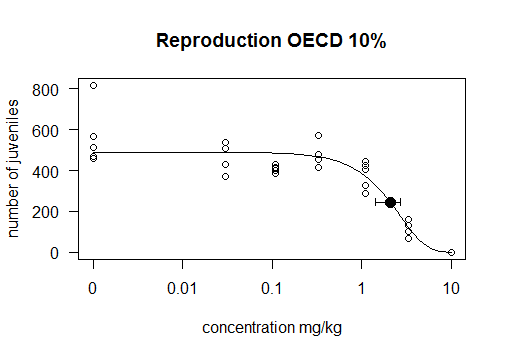


Figure S4: Dose-response relationships for the effect of imidacloprid on the survival (Top) and reproduction (Bottom) of *Folsomia candida* exposed in OECD 10 soil for 33 days


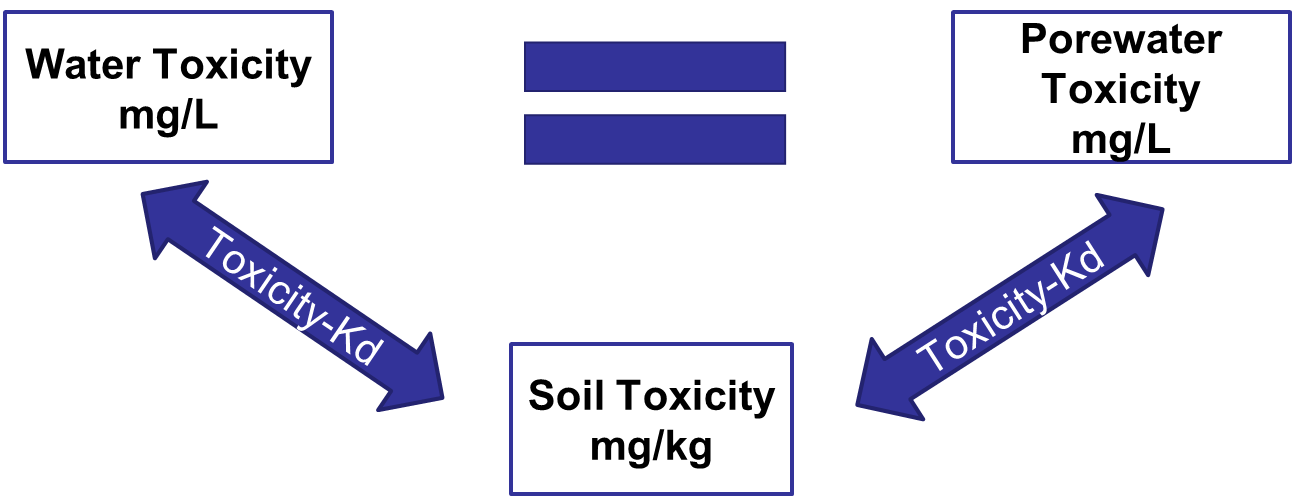


Figure S5: Schematic representation of the proposed ‘pore-water extrapolation concept’

Table S1: Lethal-moribund effect concentrations (LMC_50_) for the effect of imidacloprid on *Folsomia candida* exposed for 33 days in water or in pore water extracted from spiked LUFA 2.2 soil. 95% Confidence intervals are given in parentheses. Also given are estimated porewater LMC_50_ values in mg/L; these values were obtained from a comparison of porewater LMC_50_s in mg/kg and water LMC_50_s in mg/L (see Figure 1)

| Time (days) | Water LMC_50_  (mg/L) | Pore-water LMC_50_ (mg/kg) | Predicted Porewater LMC_50_ (mg/L) |
| --- | --- | --- | --- |
| 1 | 27.9 (22.8 - 32.9) | - | - |
| 2 | 26.1 (18.1 - 33.9) | 7.40 (3.33 - 11.5) | 75.5 |
| 3 | 26.1 (18.1 - 33.9) | 6.29 (2.53 - 10.1) | 62.5 |
| 4 | 23.2 (17.0 - 28.4) | 3.35 (2.19 - 4.50) | 27.9 |
| 5 | 23.2 (17.0 - 28.4) | 3.05 (2.01 - 4.10) | 24.4 |
| 6 | 22.4 (15.9 - 28.8) | 2.52 (1.56 - 3.48) | 18.1 |
| 7 | 16.2 (12.4 – 20.0) | 2.41 (1.12 - 3.71) | 16.8 |
| 8 | 16.2 (12.4 – 20.0) | 2.11 (0.86 - 3.63) | 13.3 |
| 9 | 15.2 (12.3 – 18.0) | 2.11 (0.86 - 3.63) | 13.3 |
| 10 | 12.6 (10.7 - 14.4) | 2.11 (0.86 - 3.63) | 13.3 |
| 11 | 12.1 (10.4 - 13.8) | 2.11 (0.86 - 3.63) | 13.3 |
| 12 | 10.9 (8.36 - 13.4) | 1.94 (0.82 - 3.05) | 11.3 |
| 13 | 10.9 (8.36 - 13.4) | 1.94 (0.82 - 3.05) | 11.3 |
| 14 | 9.99 (9.35 - 10.6) | 1.79 (1.27 - 2.33) | 9.53 |
| 33 | 3.04 (2.27 – 3.82) | 1.2 (0.70 – 1.71) | 2.59 |
